# Performance of the FACT-GOG-Ntx to assess chemotherapy-induced peripheral neuropathy (CIPN) in pediatric high risk Hodgkin lymphoma: report from the Children’s Oncology Group AHOD 1331 study

**DOI:** 10.1186/s41687-023-00653-0

**Published:** 2023-11-10

**Authors:** Susan K. Parsons, Angie Mae Rodday, Qinglin Pei, Frank G. Keller, Yue Wu, Tara O. Henderson, David Cella, Kara M. Kelly, Sharon M. Castellino

**Affiliations:** 1 Institute for Clinical Research and Health Policy Studies and Tufts Cancer Center, Tufts Medical Center, 800 Washington Street, Boston, MA 02111 USA; 2 Department of Biostatistics, Children’s Oncology Group, Statistics and Data Center, University of Florida, 2004 Mowry Rd, Gainesville, FL 32610 USA; 3 Department of Pediatrics, Emory University School of Medicine; Aflac Cancer and Blood Disorders Center, Children’s Healthcare of Atlanta, 1405 Clifton Rd, Atlanta, GA 30322 USA; 4 Department of Pediatrics, University of Chicago Pritzker School of Medicine, Comer Children’s Hospital, 5721 S Maryland Ave, Chicago, IL 60637 USA; 5 Department of Medical Social Sciences, Institute for Public Health and Medicine, Center for Patient-Centered Outcomes, Northwestern University, 420 E. Superior St, Chicago, IL 60611 USA; 6 Department of Pediatrics, Roswell Park Comprehensive Cancer Center, University at Buffalo Jacobs School of Medicine and Biomedical Sciences, 665 Elm St, Buffalo, NY 14203 USA

**Keywords:** Chemotherapy-induced peripheral neuropathy, Patient-reported outcomes, Pediatric HL

## Abstract

**Background:**

Chemotherapy-induced peripheral neuropathy (CIPN) is an under-recognized complication of several chemotherapy agents used as part of curative-intent therapy for Hodgkin Lymphoma (HL). In the absence of validated self- or proxy-report measures for children and adolescents, CIPN reporting has relied on clinician rating, with grading scales often restricted to severe manifestations. In a proof-of-concept study, we assessed the feasibility and psychometric performance of the Functional Assessment of Cancer Therapy-Gynecologic Oncology Group-Neurotoxicity (FACT-GOG-Ntx), a unidimensional CIPN symptom scale widely used adults with CIPN, in pediatric HL at risk for CIPN.

**Methods:**

Youth (11+ years) and parents of all children (5–17.9 years) with newly diagnosed high-risk HL enrolled on Children’s Oncology Group AHOD1331 (NCT02166463) were invited to complete the FACT-GOG-Ntx and a health-related quality of life (HRQL) measure at pre-treatment (Time 1), and during cycles 2 (Time 2) and 5 (Time 3) of chemotherapy during the first half of study accrual. Clinical grading of CIPN by providers was also assessed using the Balis Pediatric Neuropathy Scale. We evaluated Cronbach’s alpha, construct validity, and agreement between raters. Change in FACT-GOG-Ntx scores over time was assessed using a repeated measures model.

**Results:**

306 patients had at least one completed FACT-GOG-Ntx with time-specific completion rates of > 90% for both raters. Cronbach’s alpha was > 0.7 for youth and parent-proxy report at all time points. Correlations between FACT-GOG-Ntx and HRQL scores were moderate (0.41–0.48) for youth and parent-proxy raters across all times. Youth and parent-proxy raters both reported worse FACT-GOG-Ntx scores at Time 3 for those who had clinically-reported CIPN compared to those who did not. Agreement between raters was moderate to high. Compared to baseline scores, those at Time 3 were significantly lower for youth (β = − 2.83, *p* < 0.001) and parent-proxy raters (β = − 1.99, *p* < 0.001).

**Conclusions:**

High completion rates at all time points indicated feasibility of eliciting youth and parent report. Psychometric performance of the FACT-GOG-Ntx revealed acceptable reliability, evidence of validity, and strong inter-rater agreement, supporting the use of this self- or proxy-reported measure of CIPN in youth with high-risk HL exposed to tubulin inhibitors, as part of a Phase 3 clinical trial.

*Clinical trial information*: Clinical Trials Registry, NCT02166463. Registered 18 June 2014, https://clinicaltrials.gov/ct2/show/study/NCT02166463

## Introduction

Chemotherapy-induced peripheral neuropathy (CIPN) has been associated with several widely used chemotherapy regimens and is often under-recognized [1]. CIPN involves three principal manifestations: sensory; motor; and autonomic [2]. Manifestations vary by drug class, cumulative exposure, and genetic predisposition [3, 4]. CIPN has been shown to have negative effects on health-related quality of life (HRQL) in several disease populations. [5–7]

CIPN reporting in children and adolescents with cancer has historically relied on provider report using clinical toxicity grading scales, including the Common Terminology Criteria for Adverse Events (CTCAE) [8]. More recently, trials have also used the Balis Pediatric Scale of neuropathy [9], which is similar to CTCAE; both rely on clinician appraisal of the patient by examination and/or history. These approaches have been found to have variable degrees of inter-rater reliability, a narrow grading range, limited responsiveness to change, and substantial floor effects [2, 9]. To date there have been no validated pediatric patient-reported measures of CIPN. In contrast, the Functional Assessment of Cancer Therapy-Gynecologic Oncology Group-Neurotoxicity symptom scale (FACT-GOG-Ntx), a validated 11-item self-report measure of CIPN, has been used to measure patient self-reported CIPN in several adult cancers [10, 11]. It has been shown to have strong psychometric properties, including responsiveness to change over time [12–16].

Despite the use of vinca alkaloids in many pediatric Hodgkin Lymphoma (HL) regimens, the incidence, prevalence and trajectory of CIPN has not been extensively studied in children with HL [17]. In recent studies in adult HL patients, CIPN was recognized as a side effect of both vinblastine and Brentuximab vedotin (BV), a novel anti-CD30 antibody linked to the anti-microtubular agent, monomethyl auristatin-E (MMAE). MMAE binds to tubulin and prevents microtubule assembly, leading to cell cycle arrest and cell death. Both vinca alkaloids and BV interfere with axonal polymerization. In the 2013 report of a Phase 1 dose escalation study of BV in adult patients with newly diagnosed HL, 75% of patients developed CIPN, although most of these events were Grade 1 or 2 and manageable by dose modification [18]. This compares to rates of vincristine-induced peripheral neuropathy of 35–45% across all grades [19]. In the recent AHOD0831 and AHOD0031 protocols from the Children’s Oncology Group (COG), < 5% of pediatric/adolescent patients developed Grade 3 or higher vincristine-induced peripheral neuropathy, based on clinical grading scales. Rates of mild to moderate CIPN (≤ Grade 2) were not routinely collected [20, 21], and validated tools to measure patient report of CIPN have been lacking [22] in younger patients. While younger children may not possess the vocabulary with which to describe their symptoms, we posit that older children and adolescents are likely to be capable of providing self-report, using existing validated measures.

In a proof-of-concept study using youth and parent-proxy report, we assessed the psychometric properties of the FACT-GOG-Ntx in a Phase 3 clinical trial of pediatric patients with previously untreated high-risk HL, in which tubulin inhibitors were used as part of multi-agent therapy for high-risk HL; we compared rates of CIPN to those obtained from serial clinician grading as well as between raters (youth vs. parent).

## Methods

### Patients

Children and adolescents 2–21 years of age at diagnosis with classic HL were eligible to enroll in COG AHOD1331 (NCT02166463), a prospective randomized Phase 3 multi-center study [23]. Eligibility included newly diagnosed high-risk classic HL, defined as Ann Arbor stages IIB with bulk, IIIB or IV (A or B). Patients were not eligible if they had nodular lymphocyte predominant HL, were pregnant, had a known immunodeficiency, or had received systemic corticosteroids within 28 days of enrollment. Patients 11 years of age and older at enrollment and parents of children 5–17.9 were also invited to participate in a patient-reported outcomes (PRO) pre-specified secondary study aim, which included the serial completion of study measures. PRO collection was not an eligibility criterion for the trial participation. The planned enrollment on the PRO study, completed by September 8, 2017, was restricted to the first half of the trial accrual. Of note, the trial originally was designed for patients up to 18.9 years of age, but in August 2017, the upper age limit was amended to 21.9 years. Based on closure of recruitment to the PRO study in September 2017, only a small number of participants over 18 years were eligible.

### Treatment protocol

Patients were randomized (1:1) to receive 5 cycles of chemotherapy on one of 2 study arms, both of which included a tubular inhibitor (vincristine), along with doxorubicin, bleomycin, etoposide, prednisone and cyclophosphamide (Arm 1: ABVE-PC), or the same chemotherapy backbone with the addition of BV and the absence of bleomycin (Arm 2: BV-AVE-PC). Vincristine was administered at 1.4 mg/m2/dose (max 2.4 mg/dose) on days 1 and 8 on Arm 1, and only on day 8 of each treatment cycle in the experimental arm. BV was given at 1.8 mg/kg (maximum dose 180 mg) on day 1 of each cycle only on Arm 2. The protocol contained specific guidelines for dose modification for each of the tubulin toxins in the setting of emergent CIPN, described elsewhere [24].

The protocol was reviewed and approved by the National Cancer Institute (NCI) and the Pediatric Central Institutional Review Board (cIRB) and the local IRBs per institutional policies of participating sites. Written informed consent from parents/guardians and child assent was obtained in accordance with the Declaration of Helsinki.

### Study measures

Age-eligible youth and parent-proxy raters completed study measures at the time of study entry prior to any therapy (“baseline”) and serially through therapy and follow-up. Of note, parent proxies were not asked to project how they thought their child would rate CIPN symptoms, but rather, based their ratings on their own observations. Results collected at baseline and the two on-treatment assessments are summarized in this report. Given the exploratory use of the measure in younger children, patients aged 5–10.9 years were assessed only by their parent proxy raters, while patients aged 18–21.9 were only assessed by the youth themselves. The demographics assessment was completed at baseline by the participating parent. For each measure where dual rating was collected, there was a ‘youth’ version and a parent-proxy version. Instruments were available in English and in Spanish.

All measures were completed on paper, primarily at the time of regularly scheduled clinic visits. Completed measures were de-identified by the site clinical research assistant and then scanned into the Medidata Rave data management system, used by COG. The questionnaire was then downloaded by a study-specific research assistant, who entered responses directly into a Research Electronic Data Capture (REDCap) [25, 26] database for further analysis.

### CIPN assessment

The 11-item FACT-GOG-Ntx was collected serially prior to therapy initiation (Time 1), and again, on day 8 of cycles 2 (Time 2) and 5 (Time 3). The rationale for assessment at two on-treatment time points was to capture early onset CIPN (Time 2) and the pattern of change or worsening over time at the peak cumulative dose for the tubulin inhibitors by cycle 5. The Time 3 assessment was designed to capture the anticipated peak of CIPN temporally associated with the dose density peak exposure of both BV and vincristine.

The voice of the measure (“I” to “my child”) was modified for parent-proxy reporting in collaboration with instrument author (DC). Item wording was not otherwise changed to allow for comparisons with other studies. The preferred timing of assessment was coincident with an in-person clinic visit to enable concurrent reporting of clinical grading of neuropathy by the clinician. The measure’s item content is presented in Appendix Table 6.

The FACT-GOG-Ntx utilizes a 5-point, Likert-style response scale for each item with a one-week reference period. The FACT-GOG-Ntx is a unidimensional scale, yielding a total symptom score [12, 27], ranging from 0 to 44 in which higher scores indicated better functioning (less impairment). In practice, several investigators have also used a “short form” version of the measure, restricted to four items about sensory neuropathy [27]; sensory subscale scores ranged from 0 to 16. At least half of the items in the scale needed to be completed for it to be scored (50% rule). Previous studies have demonstrated that a change of 1/3 standard deviations (SD) on the total score [28] and a 1-point change on the sensory subscale score was clinically significant. [27]

### Clinical grading

Clinical grading of CIPN was captured at each treatment cycle with mandated reporting of ≥ Grade 2 sensory or motor neuropathy using the Balis Pediatric Neuropathy Scale [9]. In addition to specific grading, CIPN was dichotomized as absent or present at each time point.

### HRQL

The HRQL consequences of CIPN were assessed by youth and parent-proxy raters with the rater-specific version of the Child Health Ratings Inventories (CHRIs)-Global quality of life measure at each of the planned assessments [29]. We anticipated that patients with CIPN would have more diminished HRQL than those without CIPN. The CHRIs utilize a 5-point, Likert-style response scale for each item with a one-week reference period. Scores are scaled from 0–100, in which higher scores connote better functioning; at least half of the items in the scale needed to be completed for it to be scored (50% rule).

### Statistical analysis

Completion of PROs over time by rater were reported across all enrolled patients included in the PRO cohort. We described the full study population of AHOD1331 and then separately introduced those enrolled and not enrolled in the PRO cohort. Chi-square tests were used to compare the characteristics between the two groups. Mean scale scores and SDs were calculated for the 11-item FACT-GOG-Ntx, 4-item FACT-GOG-Ntx sensory subscale (FACT-GOG-Ntx-4), and CHRIs-Global by time point and rater, using established scoring algorithms.

The psychometric performance of the FACT-GOG-Ntx and FACT-GOG-Ntx-4 scales was evaluated among youth and proxy raters at the three time points (unless otherwise specified). Internal consistency reliability was calculated as Cronbach’s alpha. The minimum acceptable criterion for Cronbach’s alpha is 0.70 [30]. To assess construct validity, we calculated correlations between the FACT-GOG-Ntx, FACT-GOG-Ntx-4, and CHRIs-Global. Correlations < 0.29 were considered small, 0.3–0.59 moderate, and > 0.6 strong. Construct validity was also assessed by comparing FACT-GOG-Ntx and FACT-GOG-Ntx-4 at Time 3 by whether or not the patient had any clinically-reported CIPN (defined as > 0 score on Balis) by Time 3; two-sample t-tests were used to determine statistical significance.

Agreement between youth and parent-proxy report of the FACT-GOG-Ntx at all time periods was calculated using established methods [31]. Between-subject variation assessed the agreement between the youth and parent-proxy assessment of the patient compared to the group average. Strong correlations of between-subject variation indicate both observers consistently identified the patient as having higher or lower scores than the average patient. Within-subject variation assessed the agreement between youth and parent-proxy assessment of the patient over time. The intraclass correlation coefficient (ICC) was calculated to measure the correlation of scores within observer within time. These correlations were estimated using mixed effect models; 95% confidence intervals were estimated using 500 bootstrap samples. This analysis was restricted to patients who were eligible to have both youth and parent-proxy report (youth aged 11 to 17.9 years). Agreement between youth and parent-proxy report was also calculated separately for patients with any clinician-graded CIPN by Time 3 on the Balis scale.

To assess change in FACT-GOG-Ntx scores over time, we fit a repeated measures model (proc mixed). This analysis was restricted to patients who were eligible to have both youth and parent-proxy report (youth aged 11–17.9 years). The model included assessment time, which was treated as categorical to allow for non-linearity, rater (youth or parent-proxy), continuous youth age in years, and gender (male or female). These baseline demographic variables were selected based on their hypothesized relationship with PROs. Interactions between rater and time and rater and youth age were also assessed and included in the model if *p* < 0.1. This model assumes that data are missing at random. Unstructured, autoregressive, and compound symmetry covariance structures were considered and selected based on convergence and the lowest Akaike Information Criteria; unstructured had the best fit. Model assumptions, including normality of residuals and linearity of continuous variables, were assessed with plots of residuals.

Analyses were done using SAS software Version 9.4 for Windows (SAS Institute Inc., NC, USA) and a two-sided alpha of 0.05 was used.

## Results

### Study sample

From March 19, 2015, to September 8, 2017, 309 patients were age-eligible for the PRO study (Appendix Fig. 2). At baseline, 300 (97.1%) of 309 eligible patients or parent-proxies completed at least one PRO measure; completion of PRO measures remained high across all assessment times (> 90%) (Appendix Table 7). The FACT-GOG-Ntx was completed at least once over the three time points by 306 patients or parent-proxies. Baseline patient demographic and disease characteristics of the PRO cohort are detailed in Table 1; patients had a mean age of 15 years (SD = 2.7) and 50.5% were female. Demographic and disease characteristics of the participants enrolled in the PRO cohort were not significantly different from subsequent trial enrollees, who were not part of the PRO Cohort (Appendix Table 8).

**Table 1:** Baseline patient demographic and disease characteristics of PRO cohort

	PRO Cohort, n = 309
Age in years, mean (SD)	14.99 (2.74)
Categorical age in years, n (%)	
5 to 10.9	29 (9.39)
11 to 17.9	257 (83.17)
18–21.9	23 (7.44)
Sex, n (%)	
Female	156 (50.49)
Male	153 (49.51)
Race/Ethnicity, n (%)	
Hispanic	54 (17.48)
Non-Hispanic Black	31 (10.03)
Non-Hispanic White	189 (61.17)
Other and Unknown	35 (11.33)
Histology, n (%)	
Nodular sclerosis	240 (77.67)
Mixed cellularity	13 (4.21)
NOS	52 (16.83)
Lymphocyte-rich	4 (1.30)
Stage	
Stage IIB with Bulk	67 (21.68)
Stage III	63 (20.39)
Stage IVA	76 (24.60)
Stage IVB	103 (33.33)

FACT-GOG-Ntx scores range from 0-44, while the FACT-GOG-Ntx-4 Sensory Subscale Score range from 0-16. Higher scores on both scales indicates less symptoms. Youth report is for patients aged 11-21.9 years; parent proxy report is for youth 5 to 17.9 years

### Psychometrics of the FACT-GOG-Ntx and FACT-GOG-Ntx-4

**Fig. 1:**
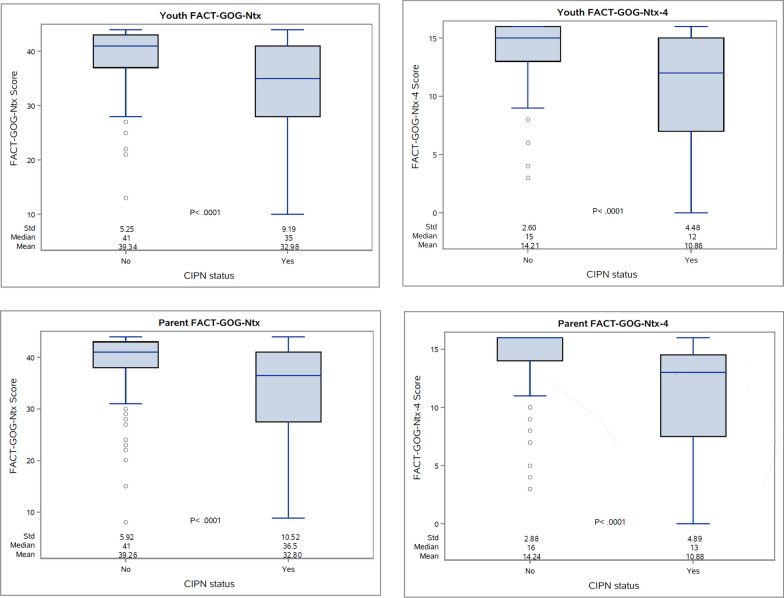
Comparison of FACT-GOG-Ntx and FACT-GOG-Ntx-4 scores by CIPN status at time 3 by rater

By Time 3, 20.1% of patients had any clinically-reported CIPN. As expected, at Time 3, FACT-GOG-Ntx and FACT-GOG-Ntx-4 scores were lower for those who had clinically-report CIPN compared to those who did not; this was significant for youth and parent-proxy rater (Fig. 1, Appendix Table 9). The mean score agreement of parents to youth reports for the FACT-GOG-Ntx was very strong across all time points (Table 2), while mean CHRIs-Global scores were consistently higher for youth than parent-proxy raters. Cronbach’s alphas were > 0.7 at baseline for youth and parent-proxy FACT-GOG-Ntx and were > 0.8 at Time 2 and Time 3 (Table 3). Over time, correlations between the FACT-GOG-Ntx and CHRIs-Global for youth raters (0.41 to 0.45) and parent-proxy raters (0.42–0.48) were moderate (Table 4).

**Table 2:** FACT-GOG-Ntx and CHRIs-global mean summary scores by rater over time

Mean (SD), n	Time 1 (Baseline)	Time 2 (D8, Cycle 2)	Time 3 (D8, Cycle 5)
CIPN			
FACT-GOG-Ntx, mean (SD)			
Youth	40.6 (4.2), n = 266	39.6 (4.9), n = 265	37.9 (6.9), n = 255
Parent-Proxy	40.9 (3.9), n = 280	40.2 (4.6), n = 278	38.1 (7.5), n = 264
FACT-GOG-Ntx-4, mean (SD)			
Youth	15.3 (1.7), n = 266	14.5 (2.4), n = 265	13.5 (3.4), n = 255
Parent-Proxy	15.3 (1.8), n = 280	14.6 (2.4), n = 278	13.6 (3.6), n = 264
HRQL			
CHRIs-Global, mean (SD)			
Youth	67.6 (22.1), n = 259	71.8 (20.8), n = 258	72.2 (22.2), n = 249
Parent-Proxy	62.2 (23.8), n = 274	66.3 (21.0), n = 269	66.7 (22.6), n = 256

CIPN, Chemotherapy-induced peripheral neuropathy. FACT-GOG-Ntx scores can range from 0 to 44 with higher scores indicating less symptoms.FACT-GOG-Ntx-4 sensory subscale score can range from 0 to 16 with higher scores indicating less symptoms. CHRIs-Global scores can range from 0 to 100 with higher scores indicating better HRQL. Youth report is for patients aged 11–21.9 years; parent-proxy report is for youth aged 5–17.9 years

**Table 3:** Cronbach’s alpha for FACT-GOG-Ntx and FACT-GOG-Ntx-4 by rater over time

	Time 1 (Baseline)	Time 2 (D8, Cycle 2)	Time 3 (D8, Cycle 5)
FACT-GOG-Ntx			
Youth	0.76	0.81	0.88
Parent-Proxy	0.71	0.80	0.90
FACT-GOG-Ntx-4			
Youth	0.67	0.75	0.85
Parent-Proxy	0.81	0.79	0.87

Youth report is for patients aged 11 to 21.9 years; parent-proxy report is for youth aged 5 to 17.9 years

**Table 4:** Correlations between FACT-GOG-Ntx, FACT-GOG-Ntx-4, and CHRIs-Global by rater over time

	Time 1 (Baseline)	Time 2 (D8, Cycle 2)	Time 3 (D8, Cycle 5)
FACT-Ntx and CHRIs-Global			
Youth	0.43	0.41	0.45
Parent-Proxy	0.42	0.44	0.48
FACT-Ntx-4 and CHRIs-Global			
Youth	0.21	0.29	0.34
Parent-Proxy	0.25	0.33	0.37

Higher FACT-GOG-Ntx, FACT-GOG-Ntx-4, and CHRIs-Global scores indicate less symptoms/better functioning, respectively. Youth report is for patients aged 11–21.9 years; parent-proxy report is for youth aged 5–17.9 years.

### Between- and within-rater agreement

Agreement between youth and parent-proxy responses to the FACT-GOG-Ntx is summarized in Appendix Table 10. The correlations of the youth and parent-proxy report of the between-subject variation for all patients, and by subgroups of interest (age and any clinically-reported CIPN), were > 0.95. The correlations between youth and parent-responses in the within-subject variation for all patients, and by subgroups of interest, ranged from 0.55 (no CIPN) to 0.70 (with any clinically-reported CIPN). The correlation of the measures within respondent over time (ICC) was similar for the youth report (0.43–0.54) and the parent-proxy report (0.43–0.53) for all patients, and by subgroups of interest.

### FACT-GOG-Ntx scores over time

In the model assessing change over time in FACT-GOG-Ntx scores, interactions between rater and time (*p* = 0.02) and rater and youth age (*p* = 0.002) were statistically significant (Table 5). Compared to baseline FACT-GOG-Ntx scores, those at Time 3 were significantly lower for youth (β = − 2.83, *p* < 0.001) and parent-proxy raters (β = − 1.99, *p* < 0.001). Older youth age was associated with lower FACT-GOG-Ntx scores in parent-proxy raters (β = − 0.27 per year, *p* = 0.01), but not in youth raters. Males had higher scores than females (β = 1.47, *p* < 0.001).

**Table 5:** Repeated measures model results for FACT-GOG-Ntx, n = 249

	β (SE)	*p*-value
Assessment time for youth		
Time 1 (reference)		
Time 2	− 0.77 (0.48)	0.11
Time 3	− 2.83 (0.48)	< 0.001
Assessment time for parent-proxy		
Time 1 (reference)		
Time 2	− 0.61 (0.44)	0.17
Time 3	− 1.99 (0.45)	< 0.001
Age (per year) for youth	− 0.03 (0.11)	0.80
Age (per year) for parent-proxy	− 0.27 (0.11)	0.01
Youth rater^a^	− 0.33 (0.23)	0.15
Male sex	1.47 (0.35)	< 0.001

Restricted to youth and parent-proxy report of patients aged 11–17.9 years

Higher FACT-GOG-Ntx scores indicate less symptoms. Interaction between rater

and time had a *p*-value of 0.02. Interaction between rater and child age had a p-value

of 0.002.

^a^Effect of youth rater is at Time 1 for a child of age 15 years

## Discussion

In this first application of the pediatric and parent-proxy FACT-GOG-Ntx and FACT-GOG-Ntx-4, we found the measures performed well in a Phase 3 clinical trial of pediatric patients at risk for CIPN due to receipt of tubulin toxins as part of multi-agent therapy for high-risk HL. Participation at study entry was high (97%), as was retention of participants in the PRO measurement cohort throughout treatment (> 90%). We observed the lowest FACT-GOG-Ntx scores, reflecting the highest patient burden of neuropathy by Time 3, hypothesized to be the peak effect time based on the cumulative dose of tubulin inhibitor therapy.

The FACT-GOG-Ntx total scale demonstrated adequate reliability, as measured by Cronbach’s alpha, construct validity based on correlations with the CHRIs-Global measure of HRQL, and meaningful differences in scores by evidence of clinically-reported CIPN. Our findings on the psychometric performance of these scales are similar to that in adult populations [12, 27].

In the pediatric oncology setting, both youth self-report and parent-proxy report provide complementary information about the patient’s experience [31], heretofore often absent from clinical trials [32]. Among youth who are able to complete PROs on their own behalf, the parent-proxy report may be confirmatory or divergent, depending on access to information and how it is evaluated by the proxy (information or criterion variance). Our study found no statistically significant differences in FACT-GOG-Ntx scores by rater, and we found moderate to strong agreement between raters. Therefore, in future studies, collecting information from only the youth self-report may be adequate to characterize the patient experience of CIPN while limiting additional burden on the parent. Among younger children who may be unable to complete PROs on their own behalf, the parent-proxy provides the only source of information, which should be reported to the provider and thereby inform clinical grading of CIPN.

As part of our assessment of construct validity, we compared FACT-GOG-Ntx and FACT-GOG-Ntx-4 scores at Time 3 and clinically-reported CIPN by Time 3. We found statistically significant and clinically significant differences in scores as reported by both raters. For example, youth without clinical evidence of CIPN had a mean FACT-GOG-Ntx score of 39.3 compared to a mean of 33.0 among youth with clinically-reported reported CIPN. The difference of six points corresponds to > 1 standard deviation difference, which far exceeds the minimal clinically important difference of 1/3 SD previously reported for this scale [28]. In addition, FACT-GOG-Ntx scores worsened throughout the course of therapy, as the cumulative incidence of CIPN increased.

The strengths of the study include the use of an established and well-validated measure of CIPN in adults applied in an at-risk pediatric population. Serial completion rates of the PROs were high, despite initial high-risk disease and intensity of treatment, and the opt-in nature of participation. Further, the PRO cohort did not differ from the non-PRO cohort on patient- or disease-level characteristics, suggesting that study findings could be generalized to the entire study population and demonstrated the pre-determined statistical capacity to limit this sub-study to half the trial enrollment cohort.

We also acknowledge study limitations. Given the adolescent age of the study population and the measure’s simplicity of language, we elected not to conduct cognitive interviewing or replicate the underlying factor structure of the FACT-GOG-Ntx in this trial. Previous psychometric evaluation of the measure over the past 20 years supports its total score through strong item-total correlations [13, 33]. In this study, our goal was to demonstrate that it would be possible to collect patient-reported CIPN symptoms in the trial setting, setting the stage for future comparisons across age groups, such as in the recently accrued SWOG-led S1826 trial of adolescents, young adults, and older adults with newly diagnosed advanced stage HL (NCT03907488). In the S1826 study, tubulin toxins were again used; participant-reported CIPN using the FACT-GOG-Ntx was successfully collected from all participants 12 years of age and older over time. In future studies involving younger patients, however, we would encourage investigators to utilize cognitive interviewing to ensure meaning of individual items.

In conclusion, we demonstrate the feasibility and performance of the FACT-GOG-Ntx in assessing CIPN in older youth with high-risk HL, exposed to tubulin toxins. The strength of its performance suggests that this measure should be considered in future trials to characterize CIPN and its functional consequences, the latter measured by the parallel collection of HRQL. Given the lack of apparent participant burden, as evinced by high completion rates, we would recommend the use of the 11-item FACT-GOG-Ntx measure, rather than just the use of the 4-item subscale score, providing greater reliability and construct validity of CIPN among at-risk patients.

## Figures and Tables

**Fig. 2:**
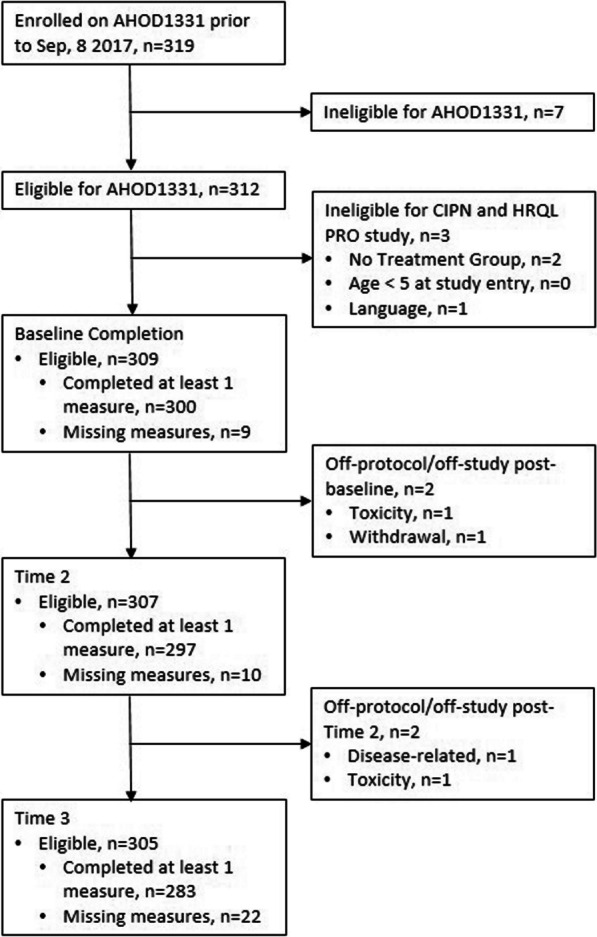
Study Schema Showing Enrollment into AHOD1331 CIPN and HRQL PRO Study and Measure Completion over Time

## Data Availability

The COG data sharing policy describes the release and use of COG’s individual subject data for use in research projects in accordance with National Clinical Trials Network Program and National Cancer Institute Community Oncology Research Program Guidelines and following the publication of the primary publication (November 2022). Requests for access to COG protocol research data should be sent to datarequest@childrensoncologygroup.org. Data are available to researchers whose proposed analysis is found by COG to be feasible and of scientific merit and who agree to the terms and conditions of use.

## Appendix

See Fig. 2

See Tables

**Table 6:** Item Content of FACT-GOG-Ntx

Numbness or tingling in hands
Numbness or tingling in feet
Discomfort in hands
Discomfort in feet
Joint pain or muscle cramps
Feel weak all over
Trouble hearing
Ringing or buzzing in ears
Trouble buttoning buttons
Trouble feeling the shape of small objects in hand
Trouble walking

Actual wording of each item can be found on the FACIT website (https://www.facit.org/measures/FACT-GOG-NTX, accessed 09/11/2023)

6,

**Table 7:** Completion of measures over time

Measure, n (%)	Time 1 (Baseline)	Time 2 (D8, Cycle 2)	Time 3 (D8, Cycle 5)
Baseline Demographics	287		
Youth FACT-GOG-Ntx	266	265	255
Parent-Proxy FACT-GOG-Ntx	280	278	264
Youth CHRIs-Global	259	258	249
Parent-Proxy CHRIs-Global	274	269	256
Any measure completed ^a^	300 (97.1%)	297 (96.7%)	283 (92.8%)
Eligible	309	307	305

^a^At least one of the following was completed at that time period: Youth FACT/GOG-Ntx, Parent-Proxy FACT/GOG-Ntx, Youth CHRIs-Global, Parent-Proxy CHRIs-Global. Youth is for patients aged 11 to 21.9 years; parent-proxy is for youth aged 5 to 17.9 years

7,

**Table 8:** Comparison between baseline patient demographic and disease characteristics of PRO cohort and non-PRO cohort

	Total, n = 587	PRO Cohort, n = 309	Non-PRO Cohort n = 278	P-value^a^
Age in years, mean (SD)	15.03 (3.10)	14.99 (2.74)	15.08 (3.46)	0.73
Categorical age in years, n (%)				0.15
2 to 4.9	4 (0.68)	0 (0.00)	4 (1.44)	
5 to 10.9	58 (9.88)	29 (9.39)	29 (10.43)	
11 to 14.9	176 (29.98)	98 (31.72)	78 (28.06)	
15 to 21.9	349 (59.45)	182 (58.90)	167 (60.07)	
Sex, n (%)				0.08
Female	276 (47.02)	156 (50.49)	120 (43.17)	
Male	311 (52.98)	153 (49.51)	158 (56.83)	
Race/Ethnicity, n (%)				0.23
Hispanic	119 (20.27)	54 (17.48)	65 (23.38)	
Non-Hispanic Black	59 (10.05)	31 (10.03)	28 (10.07)	
Non-Hispanic White	338 (57.58)	189 (61.17)	149 (53.60)	
Other and Unknown	71 (12.10)	35 (11.33)	36 (12.95)	
Histology, n (%)				0.48
Nodular sclerosis	449 (76.49)	240 (77.67)	209 (75.18)	
Mixed cellularity	33 (5.62)	13 (4.21)	20 (7.19)	
NOS	98 (16.70)	52 (16.83)	46 (16.55)	
Lymphocyte-rich	7 (1.19)	4 (1.29)	3 (1.08)	
Stage, n (%)				0.19
Stage IIB with Bulk	121 (20.61)	67 (21.68)	54 (19.42)	
Stage III	113 (19.25)	63 (20.39)	50 (17.99)	
Stage IVA	167 (28.45)	76 (24.60)	91 (32.73)	
Stage IVB	186 (31.69)	103 (33.33)	83 (29.86)	
B symptoms, n (%)				0.13
No	162 (27.60)	75 (24.27)	87 (31.29)	
Yes	418 (71.21)	231 (74.76)	187 (67.27)	
Unknown	7 (1.19)	3 (0.97)	4 (1.44)	
Bulk^b^, n (%)				0.29
No	172 (29.30)	82 (26.54)	90 (32.37)	
Yes	411 (70.02)	225 (72.82)	186 (66.91)	
Unknown	4 (0.68)	2 (0.65)	2 (0.72)	
Large mediastinal mass (LMA)^b^, n (%)				0.15
No	263 (44.80)	128 (41.42)	135 (48.56)	
Yes	323 (55.03)	180 (58.25)	143 (51.44)	
Unknown	1 (0.17)	1 (0.32)	0 (0.00)	
Bone marrow disease, n (%)				0.75
No	497 (84.67)	263 (85.11)	234 (84.17)	
Yes	90 (15.33)	46 (14.89)	44 (15.83)	

^a^*p*-values compare PRO Cohort and Non-PRO Cohort

^b^Bulk is defined as LMA (transverse tumor diameter > 1/3 the thoracic diameter at the dome of the diaphragm on a 6-foot posterior-anterior upright chest radiograph) or extra-mediastinal bulk (a continuous aggregate of nodal tissue outside the mediastinum that measures > 6 cm in transverse dimension on axial CT or longest dimension on coronal or sagittal reformatted CT)

8,

**Table 9:** Comparison of FACT-GOG-Ntx and FACT-GOG-Ntx-4 by Clinically-Reported CIPN by Time 3

Mean (SD), n	No CIPN by Time 3	CIPN by Time 3	P-value
FACT-GOG-Ntx			
Youth	39.34 (5.25), n = 197	32.98 (9.19), n = 57	< 0.001
Parent-Proxy	39.26 (5.92), n = 189	32.80 (10.52), n = 52	< 0.001
FACT-GOG-Ntx-4			
Youth	14.21 (2.60), n = 197	10.86 (4.48), n = 57	< 0.001
Parent-Proxy	14.24 (2.88), n = 189	10.88 (4.89), n = 52	< 0.001

FACT-GOG-Ntx scores can range from 0 to 44 with higher scores indicating better functioning

FACT-GOG-Ntx-4 scores can range from 0 to 16 with higher scores indicating better functioning

Youth report is for patients aged 11–21.9 years; parent-proxy report is for youth aged 5–17.9 years

9,

**Table 10:** Between and within parent and child correlations for FACT-GOG-Ntx, n = 257

	Agreement between youth and parent		Correlation of responses within respondent	
	Between-subject variation, Corr (95% CI)	Within subject variation, Corr (95% CI)	Within youth, ICC (95% CI)	Within parent, ICC (95% CI)
FACT-GOG-Ntx				
Overall	0.96 (0.962–0.967)	0.62 (0.615–0.624)	0.48 (0.473–0.481)	0.45 (0.444–0.455)
CIPN	0.99 (0.984–0.990)	0.70 (0.691–0.701)	0.48 (0.476–0.490)	0.40 (0.393–0.408)
No CIPN	0.94 (0.936–0.946)	0.55 (0.543–0.555)	0.42 (0.417–0.428)	0.41 (0.402–0.422)
Age 11–14.9 years	0.96 (0.951–0.963)	0.55 (0.539–0.559)	0.53 (0.518–0.532)	0.51 (0.494–0.516)
Age ≥ 15 years	0.97 (0.971–0.976)	0.63 (0.628–0.638)	0.46 (0.456–0.466)	0.42 (0.419–0.430)

Restricted to youth and parent-proxy report of patients aged 11–17.9 years

10.

## Abbreviations

CIPN: Chemotherapy-induced peripheral neuropathy
HRQL: Health-related quality of life
CTCAE: Common Terminology Criteria for Adverse Events
FACT-GOG-Ntx: Functional assessment of cancer therapy-gynecologic oncology group-neurotoxicity symptom scale
HL: Hodgkin lymphoma
BV: Brentuximab vedotin
MMAE: Monomethyl auristatin-E
COG: Children’s Oncology Group
PRO: Patient-reported outcomes
NCI: National Cancer Institute
SD: Standard deviation
CHRIs: Child Health Ratings Inventories
ICC: Intraclass correlation coefficient
